# Effect of Flower Storage and Harvest Times on the Dynamics of Volatile Compounds in Ylang-Ylang [*Cananga odorata* (Lam.) Hook.f. &Thomson] Essential Oil

**DOI:** 10.3390/plants15060891

**Published:** 2026-03-13

**Authors:** Abacar Chakira, Christian Soria, Jérôme Minier, Marc Chillet, Cyrielle Garcia

**Affiliations:** 1 Qualisud, Univ Montpellier, Univ La Réunion, CIRAD, Institut Agro, IRD, Avignon Univ, Montpellier, France; chakira_abacar@yahoo.fr (A.C.); jerome.minier@cirad.fr (J.M.); marc.chillet@cirad.fr (M.C.); 2 CIRAD, UMR Qualisud, F-97410 Saint-Pierre, Réunion, France; 3 LAR2SN, Faculté des Sciences et Techniques, Université des Comores, Moroni, Comoros

**Keywords:** *Cananga odorata*, ylang-ylang, post-harvest practices, essential oil, volatile compounds

## Abstract

This study examines the effects of flower storage duration and harvest time on the yield and evolution of the volatile composition of ylang-ylang (*Cananga odorata* forma *genuina*) essential oil. Oils were obtained from flowers by hydrodistillation immediately or after storage (4 h, 8 h, 24 h and 30 h) to investigate the effect of storage duration and from flowers picked at different schedules (07:00 a.m., 11:00 a.m. and 03:00 p.m.) to investigate the effect of the time of flower harvesting. The ylang-ylang oils were by analyzed by GC–MS. Storage duration had no significant effect on yield up to 8 h, whereas significant decreases occurred after 24 and 30 h. The amount of aromatic compounds decreased markedly with storage, from 73.66% in fresh petals to 57.02% after prolonged storage. Harvest time also influenced the distribution of oxygenated compounds, terpene hydrocarbons and several key volatiles. Overall, oils distilled within 0–8 h after harvest and obtained from flowers collected at 07:00 a.m. or 3:00 p.m. exhibited superior yields and chemical profiles compared with delayed distillation or midday harvest (11:00 a.m.). These results underline the importance of rapid processing and optimized harvest timing to preserve oxygenated fractions and essential-oil quality.

## 1. Introduction

*Cananga odorata* (Lam.) Hook.f. & Thomson, commonly called ylang-ylang, is well-known for its fragrant flower. The tree, native to the Moluccan island, has been largely implemented in humid tropical zones, but today it is mainly grown on islands in the Indian Ocean. The Comoros archipelago and Madagascar are the world’s leading producers of ylang-ylang essential oil, a fragrant oil of great value in the perfumery and food industries [1,2].

The chemical composition, quality and quantity of plant oils are known to vary according to several factors, such as the geographical origin of the plant, the different variants of the same species, the part of the plant used for extraction, the harvest period, the distillation method and the storage time [3,4,5]. The harvesting and post-harvesting stages are important for obtaining higher content and better quality essential oils from herbal and aromatic plants.

As harvested ylang-ylang flowers perish rapidly, post-harvest handling of the flowers prior to oil extraction is a vital step in ylang-ylang essential-oil (EO) production. However, the ylang-ylang value chain remains largely handcrafted in major producing regions such as the Comoros and Madagascar. Flowers are generally handpicked daily during early-morning hours and must be transported rapidly to distillation sites. However, transportation is often slowed by the poor condition of rural roads and limited infrastructure, meaning that several hours may elapse between harvest and distillation. These delays, combined with high ambient temperatures and the fragility of the fresh flowers, can significantly affect the biochemical integrity of the raw material and, ultimately, the chemical profile and quality of the essential oil [6].

Minimizing the delays between harvest and distillation, and using cool, aerated storage is critical to avoid deterioration of the essential-oil quality. Excessive temperatures inside bags of harvested flowers, due to their high respiration rate, results in a loss of essential-oil quantity and quality [7,8]. Several studies have shown that post-harvest storage can negatively affect essential-oil yield and quality across a wide range of aromatic crops [7,8,9,10,11].

In the Comoros, a qualitative study on the critical operations for quality management throughout the life cycle of ylang-ylang revealed a substantial variability in technical practices among producers and distillers [12]. Flower harvesting is particularly variable: although it is recommended from dawn until 10 a.m., when aromatic compounds peak due to nocturnal synthesis [1], many producers continue harvesting well into the day, exposing flowers to higher temperatures that may accelerate volatilization and metabolic degradation. This variability was also observed for post-harvest handling with geographical features. On Mohéli Island, where flower production frequently exceeds the processing capacity of available distillation units, flowers are commonly stored under shaded conditions for periods ranging from several hours to one day or more before distillation. On Anjouan Island, where distillation capacity exceeds flower availability, unit distillers often prolong flower storage beyond one day while awaiting sufficient quantities for processing [12]. These contrasting, handcrafted and non-standardized practices further justify investigating how harvest time and post-harvest storage conditions modulate the chemical dynamics and quality of ylang-ylang essential oil.

Time of harvesting and processing are important non-monetary agronomic inputs which influence essential-oil yield and composition. In the literature there is limited data available for the role of diurnal yield in the chemical composition of essential oil [13,14,15,16]. Several studies have been conducted on chemical variability of ylang-ylang essential oils; however, no study has been reported in the literature so far on the influence of the storage and harvest time of the flowers on the yield and chemical composition of the extracted oils.

The present work aimed to study the kinetic changes in the relative abundance of essential-oil volatile constituents, according to harvest and post-harvest practices. This dynamic behavior was investigated for essential oils obtained from flowers stored for different durations and harvested at different times. GC–MS analyses were performed to compare the relative abundances of individual compounds, families and subgroups of chemical.

## 2. Experiment

### 2.1. Plant Material

Fresh ylang-ylang flowers (*Cananga odorata*, forma *genuina*) were handpicked at full maturity in Saint-Pierre, Réunion Island, France, at two nearby sites (GPS coordinates: −21.27724; 55.46689 for the storage duration experiment and −21.31514; 55.48687 for the harvest time experiment). Only flowers displaying a purplish-red central corolla, corresponding to the optimum maturity stage for essential-oil extraction, were selected and immediately transported to the laboratory for processing.

To investigate the effect of storage duration prior to distillation on essential-oil quality, flowers picked at 07:00 a.m. were divided into five equal batches of 100 g each. Flowers were stored under shaded, open-air, non-compressive conditions by spreading them in thin layers on tables, allowing natural aeration. During the experimental period, the ambient temperature ranged from 23.6 °C to 33.2 °C, while the relative humidity varied between 48% (warmest hours) and 86% (cooler periods). The batches were distillated immediately (ST0) or after 4 h (ST4), 8 h (ST8), 24 h (ST24) and 30 h (ST30) of storage. The weight lost by the flowers during storage was determined by measuring their weight before and after each storage period using an analytical balance (Sartorius Entris II BCE224I-1S; Sartorius AG, Göttingen, Germany). The weight lost was expressed as the percentage of weight reduction relative to the initial mass. The experiment was conducted in triplicates between January and March 2021.

To investigate the effect of harvest time on essential-oil quality, flowers were picked at 07:00 a.m. (HT7), 11:00 a.m. (HT11) and 03:00 p.m. (HT15) in batches of 100 g each. The harvest location was located within a short walking distance away from the laboratory to minimize handling time, and the flowers were transported and distilled immediately. The experiment was conducted in triplicate between December 2019 and February 2020 at the same experimental site. Flowers were collected from the same individual tree at each harvest time in order to minimize genetic and plant-to-plant variability.

### 2.2. Essential-Oil Extraction, Yield Measurement and Volatile Compound Analysis

The harvested ylang-ylang flowers were subjected to hydrodistillation using a Clevenger-type device for flowers, with each batch of fresh petals mixed with 1.5 L of distilled water. The extraction time was 180 min (measured after the appearance of the first droplets of condensed oil).

The extraction yield of essential oils (EO) was calculated from the vegetable matter (VM), according to the following formula, EO (% *w*/*w*) = (mEO/mVM) × 100, where m is the mass expressed in grams.

The essential-oil samples were diluted in ethanol and characterized using a gas chromatography mass spectrometer (Clarus580 SQ8, PerkinElmer, Villebon sur Yvette, France) equipped with Elite-5MS fused silica capillary columns (PerkinElmer, Shelton, MT, USA) for mass spectrometry and for GC-FID. The mass spectrometer (MS) was directly coupled to the GC, allowing the simultaneous acquisition of GC and MS chromatograms following sample injection. The oven temperature program was as follows: initial temperature of 65 °C (held for 1 min), increased to 190 °C at a rate of 4 °C/min, then to 285 °C at a rate of 15 °C/min, and maintained for 2 min for both Elite-5MS columns. The injector and detector temperatures were maintained at 260 °C and 300 °C, respectively. Helium served as the carrier gas at a constant flow rate of 0.6 mL/min. The injection volume was 0.2 µL, with an MS data acquisition rate of 25,000 pts/s. For mass spectrometric detection, the ionization energy was set to 70 eV, and mass spectra were acquired within the m/z range of 30–400. The transfer line and ion source were maintained at 250 °C and 230 °C, respectively, and the electron multiplier voltage was adjusted to 1568 V. A solvent delay of 6 min was applied, with a total run time of 74.5 min per analysis. A mixture of commercial C7–-C30 n-alkanes (49451 U, Supelco, Bellefonte, PA, USA) was injected into the GC–MS instrument under the same conditions to calculate the retention indices (RIs). Compound identification was achieved using the NIST MS database 17 by comparing the mass spectra and RIs stored in the database with those obtained via the Elite-5MS column. The relative quantification was conducted based on the percentage area ratio of each compound to the total peak area of all compounds. Quantitative data are expressed as normalized relative peak areas and therefore represent relative compositional variations rather than absolute concentrations.

The retention indices (RIs) were calculated according to the linear retention index (LRI) method of Van den Dool and Kratz using a homologous series of n-alkanes (C7–C30) analyzed under the same chromatographic conditions. The RI values were determined using the following equation: RI = 100 × [n + (tR(x) − tR(n))/(tR(n + 1) − tR(n))]; where tR(x) is the retention time of the target compound, *t*R (n) and *t*R (n + 1) are the retention times of the n-alkanes eluting immediately before and after the compound, respectively, and *n* is the number of carbon atoms of the preceding n-alkane. The retention indices were calculated and recorded exclusively on the Elite-5MS (non-polar) column under the GC–MS conditions described in this section.

### 2.3. Statistical Analysis

Statistical analyses were carried out using R/R Studio Statistical Software (v4.1.0; R Core Team, 2021) within the RStudio environment, with the appropriate R packages, including Rmisc (v1.5.1), agricolae (v1.3-5), dplyr (v1.0.7), ggplot2 (v3.3.5), and additional supporting packages. The significant differences between the ylang-ylang essential oils were assessed using analysis of variance (ANOVA), followed by Fisher’s least significant difference (LSD) test at a significance level of *p* ≤ 0.05.

## 3. Results and Discussions

In this study, essential-oil quality was evaluated using several complementary criteria specific to ylang-ylang. Commercial quality is assessed based on classification into commercial fractions, which are mainly determined by oil density. Extraction yield represents an important economic parameter for producers and is considered as an added-value indicator rather than a direct criterion of essential-oil quality. Aromatic quality is evaluated according to oils rich in oxygenated compounds and relatively poor in hydrocarbon terpenes, since such profiles are generally associated with superior olfactory properties. Individual chemical composition was also determined to characterize key compounds and subgroups of chemical compounds. Interpretations are based on statistically significant and reproducible trends observed across triplicates.

### 3.1. Evolution of Flower Weight Loss and Essential-Oil Yield with Storage Duration

As presented in Table 1, the duration of flower storage under shaded open-air conditions markedly influenced both the weight lost and the essential-oil yield from the ylang-ylang petals. A gradual and significant (*p* < 0.01) increase in petal weight loss was observed as storage time progressed, particularly between ST0 and ST4 and between ST8 and ST24. This trend indicates that prolonged post-harvest exposure to ambient air, even under shaded conditions, promotes substantial evaporation from the flowers.

The weight loss observed during storage mainly reflects the evaporation of water from the flowers. Changes in essential-oil composition are therefore more likely associated with physiological and physicochemical processes occurring during storage, such as volatilization of highly volatile compounds or oxidative transformations.

Physiologically, once detached, flowers remain metabolically active; respiration and transpiration drive water loss and carbon depletion, accelerating tissue desiccation and reducing recoverable volatiles. Lowering the temperature slows these processes (and the ethylene action), whereas ambient storage accelerates them, shortening the window for optimal extraction [17]. Comparable dynamics are well documented in other perfume crops. In Damask rose, EO yield and composition degrade over time under ambient conditions or during extended fermentation and storage: oils distilled immediately after harvest show higher yields than those processed 12–36 h later, with progressive losses thereafter. Time and temperature management (rapid processing or chilled storage) mitigates both mass loss and compositional drift. The fact that the ST0–ST8 yields remained statistically similar, while significant decreases appear after 24 h or more, is fully consistent with these previous findings [7]. In this study, flowers were stored under shaded, aerated, and non-compressive conditions by spreading them on tables. This approach limited mechanical damage, friction, and overheating, which are major factors promoting cellular breakdown and volatile losses in flowers. Therefore, the observed storage effects likely represent conservative estimates compared with typical field practices involving bagging or piling.

From a quality perspective, these physiological processes have direct consequences on oil recovery. Water loss concentrates tissues but does not translate into higher EO recovery; instead, prolonged storage favors volatile loss and biochemical shifts.

The yield losses obtained for ST0, ST4 and ST8 are close but significantly higher compared to those obtained for ST24 and ST30 (Table 1). During the first hours of storage, flowers may experience a short physiological stabilization phase. Moderate water loss (<30%) could concentrate intracellular metabolites without yet triggering significant metabolic degradation, thus maintaining comparable oil yields to freshly distilled petals. Similar transient equilibria have been observed in *Rosa damascena* during the first 6–8 h post-harvest, when mild dehydration slightly concentrates volatiles without altering biosynthetic enzyme activity [7,10].

In our study, beyond 8 h of storage, progressive oxygen depletion and increased internal temperature inside flower piles may create micro-anaerobic conditions, favoring fermentation processes. These conditions enhance enzymatic hydrolysis and the oxidation of volatile precursors, leading to losses of key compounds and reduced oil recovery [18,19]. The decline observed at ST24 and ST30 thus likely reflects the cumulative effects of respiration, ethanol fermentation, and volatilization of labile aroma compounds (Table 1). Prolonged storage also causes mechanical and oxidative deterioration of glandular trichomes, the main sites of essential-oil accumulation. Inan et al. showed that increased environmental temperature and respiration-driven heating during storage can rupture these structures, allowing partial evaporation or oxidation of stored terpenes [5]. This would explain the sharp yield decline after 24 h. Finally, microbial proliferation on moist petals under ambient conditions can intensify hydrolytic and oxidative reactions that degrade volatile esters and phenolics [20]. This biochemical destabilization further contributes to the irregular kinetic profile observed.

As shown in Table 1, essential-oil yield remained relatively stable between ST0 and ST8 (0.78–0.76%), whereas a significant decline was observed after prolonged storage, with values decreasing to 0.73% and 0.72% at ST24 and ST30, respectively, in parallel with increasing flower weight loss. A similar observation was reported for rose flower distillation. For instance, the oil content in the flowers distilled immediately after the harvest was 0.035% and was only 0.03% 12 h later, 0.027% 24 h later and 0.025% 36 h later [7].

From an economic perspective, the decrease in essential-oil yield observed between ST4 and ST30 represents a relative reduction of approximately 2.6%, or even 7.7% between ST0 and ST30 if distillation capacity and distance from harvest sites allow for rapid processing. Considering the total production of ylang-ylang essential oil in Madagascar and the Comoros (approximately 110 tonnes per year), such a reduction would represent a loss of between 2.3 and 7.7 tonnes per year. These figures highlight that even moderate variations in yield can have significant economic consequences on an industrial scale.

### 3.2. Evolution of Volatile Compounds Along Storage

The effects of storage duration on individual aromatic compounds are presented in Table 2. Storage time affected the main aromatic constituents, in the subgroups of esters, phenols and ether oxides, although with contrasting response patterns depending on the compound considered. Indeed, benzyl acetate, methyl benzoate, benzyl benzoate, p-cresyl methyl ether and benzyl salicylate showed comparable concentrations during short storage durations (ST0, ST4 and ST8), followed by a significant change after prolonged storage, with ST24 and ST30 displaying similar values. In contrast, p-cresol and benzeneacetaldehyde exhibited an earlier sensitivity to storage, with significant differences observed between fresh flowers (ST0) and prolonged storage durations (ST24 and ST30), while intermediate storage times showed no marked variation. Ethyl benzoate displayed a distinct progressive pattern, with similar levels between ST0 and ST4, a significant increase at ST8, and further significant changes at ST24 and ST30, which remained statistically similar.

From an olfactory perspective, the aromatic esters benzyl acetate, methyl benzoate, ethyl benzoate, and benzeneacetaldehyde are among the primary contributors to the bright floral fruity and top notes of ylang-ylang essential oil. Benzyl acetate is classically described as sweet, fruity and jasmine-like, while methyl and ethyl benzoate provide narcotic floral and sweet–fruity nuances also associated with ylang-ylang and tuberose [19,21]. Benzeneacetaldehyde brings strong green floral notes and hyacinth and honey-like aromas, essential to fresh floral accords [22]. In contrast, benzyl benzoate and benzyl salicylate exhibit mild sweet balsamic, oily herbal odors and are mainly used as fixatives, reinforcing warmth, roundness and persistence in floral compositions [21]. Phenolic derivatives such as p-cresyl methyl ether and p-cresol contribute to narcissus-like, animalic or medicinal notes that strongly shape floral depth, as similarly reported in volatile profiling studies of fermented or fruity products [23]. Therefore, the decline in floral fruity esters related to storage should reduce the freshness and brightness of the top notes, whereas changes in benzenoid fixatives and phenolic derivatives should mainly affect the depth, warmth, and overall balance of the dry down.

Similar observations made of the main aromatic compounds (phenols) of *Thymus daenensis* reported that the contents of carvacrol and thymol increased in all treatments depending on the storage time of the flowers, particularly at room temperature [24]. For linalool, one of the very odoriferous compounds of ylang-ylang essential oils, the results showed no statistically significant variation during storage, similarly to *Thymus daenensis*. where it remains stable for 2 and 3 months of storage. The main components of *Rosa damascene* (geraniol, nerol and citronellol) were also reported to be significantly influenced by flower storage durations up to 4 days [25]. A decreasing trend with increasing storage duration was observed for contents of benzyl acetate, p-cresyl methyl ether, methyl benzoate, prenyl acetate and 3-methyl-3-butene-1-ol acetate (Table 2). In contrast, benzyl salicylate, α-farnesene, germacrene-D, benzyl benzoate, guaiol and δ-cadinene exhibited an opposite pattern, with their relative abundances increasing as storage duration progressed. For most of these compounds, no significant differences were detected among short storage durations (ST0, ST4 and ST8), whereas their contents were significantly lower or higher after prolonged storage (ST24 and ST30), which displayed statistically similar values. Among the aromatic compounds significantly influenced by the storage period, we found chavicol was detected only in oil from unstored flowers (ST0) (0.13%); N-carbobenzyloxy-l-tyrosyl-l-valine, cinnamaldehyde (E)- and benzaldehyde were absent in oils from unstored flowers but their content was rather similar at the other storage times (ST4, ST8, ST24 and ST30); and prenyl benzoate was not detected at ST0 and ST4 but was fairly similar in content at ST8, ST24 and ST30.

Terpene compounds are the most significantly influenced by flower storage duration. α-farnesene, germacrene-D, copaene, δ-cadinene, farnesol and guaiol all increased from ST0 to ST30. Other terpene compounds are also influenced by storage time, among them: epi-cubebol and *β*-myrcene are identified only at ST0; ylangene and trans-nerolidol at ST4; germacrene-D-4-ol at ST30; and others (Table 2). An increase of oxygenated terpenes was also observed in *Rosa damascena* during storage [25]. Small increasing trends in the contents of germacrene-D (0.34%, 0.48, 0.63%) and farnesol were reported (nd, 0.18% and 0.20%) from 0, 10 and 20 days of storage duration, respectively [26].

Prenyl acetate, 3-methyl-3-butene-1-ol acetate, 2-hexanal, (Z) and (E,E)-farnesyl acetate are the aliphatic derivative compounds most influenced by the storage period of ylang-ylang flowers. All but (E,E)-farnesyl acetate, which has an opposing trend, decreased with storage. With the exception of *trans*-2-hexanal, which showed a significant difference between ST0 and ST24–ST30, no significant differences were observed among ST0, ST4 and ST8, nor between ST24 and ST30 for the other compounds. Among the aliphatic derivative compounds, butyl acetate has specific changes at ST0; prenol at ST8, ST24 and ST30; and 2-methyl-4-(2,6,6-trimethylcyclohex-1-enyl)but-2-en-1-ol and cyclopentanone, 3-[3, 5-decadienyl]-, (Z, Z)- at ST24 and ST30 (Table 2).

### 3.3. Evolution of Chemical Compound Subgroups During Storage

**Figure A1:**
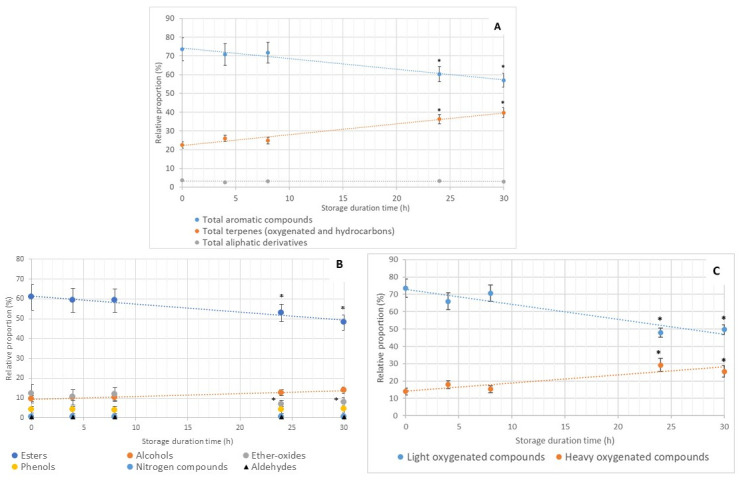
Evolution of chemical compound family and subgroup proportion during storage period of ylang-ylang flowers. (**A**)—families of chemical compounds, (**B**)—subgroups of chemical compounds, (**C**)—oxygenated compounds. Values are presented as me ± SD, n = 3. A significant difference with immediate distillation for the same family of chemical compounds is indicated with an asterisk: *, *p* < 0.05.

The results clearly show that the storage duration influenced the composition of the ylang-ylang essential oil. Significant changes in the proportion of chemical compound families were observed, such as in the total aromatic compounds and total terpenes during storage, whereas there was no effect on the total aliphatic derivates (Figure A1A). The total content of aromatic compounds decreased during storage from ST0 to ST30. Thus, the relative proportion of the aromatic compound family was almost similar between the short storage times (ST0, ST4, and ST8), but these values were significantly higher compared to the longer storage durations (ST24 and ST30). In parallel, the total terpenes content increased as the storage duration increased. The proportion of terpene family compounds was similar between the short storage times (ST0, ST4, and ST8), but these values were significantly lower compared to ST24 and ST30. Thus, in this study, significant differences are generally observed from 24 h of flower storage duration.

Total oxygenated compounds represent approximately 82% of the identified volatile compounds and were also found to be influenced by the storage duration (Figure A1B). Among them, light oxygenated compounds characterized by a low molecular weight (typically <150 g/mol), high volatility/low boiling point, and simple functional subgroups such as esters, alcohols, aldehydes, and ketones, are the most abundant and had higher contents in unstored petals (ST0) than in stored petals at ST24 and ST30. On the contrary, heavy oxygenated compounds characterized by high molecular weight (generally >150 g/mol), low volatility/high boiling point, and complex structures such as sesquiterpene alcohols, oxides, or lactones, increased from ST8 to ST24 (Figure A1C). For these two oxygenated compound categories, the values observed for ST0, ST4 and ST8 are rather similar but significantly different to the values observed at ST24 and ST30, which are close. Overall, the content of total oxygenated compounds decreased as the storage time increased and this decrease is very significant between ST0 and ST24 (Figure A1).

Oxygenated compounds, particularly esters (e.g., benzyl acetate), alcohols (e.g., linalool), and ether oxides (e.g., p-cresyl methyl ether), play a central role in determining the olfactory quality of ylang-ylang essential oils by contributing to their characteristic floral, sweet, and balsamic notes [27,28,29]. These compounds largely govern aroma intensity and sensory appreciation, whereas hydrocarbon terpenes have a limited impact on fragrance perception [30].

Previous studies have shown that the total oxygenated compounds increase with flower maturity and are closely associated with superior organoleptic properties and higher commercial value [6,31]. In addition to their sensory role, several oxygenated constituents exhibit biological activities, including antimicrobial and antioxidant effects, supporting their use in cosmetic, pharmaceutical, and wellness applications [30]. Furthermore, optimized extraction methods have been shown to improve the preservation of oxygenated compounds, thereby enhancing overall essential-oil quality [32].

The magnitude of the compositional shifts observed after 24–30 h of storage is consistent with post-harvest dynamics previously reported in other essential-oil crops. In *Rosa damascena*, ambient storage (12–36 h) induces a pronounced decline in oxygenated monoterpenes coupled with an increase in hydrocarbon terpenes, reflecting fermentation, volatilization of esters and oxidative degradation during senescence [7,10,26]. Comparable trends have also been reported for citrus blossoms: in neroli oil from bitter orange flowers, prolonged storage reduces oxygenated monoterpenes while promoting the formation of oxidized derivatives that progressively dull the floral freshness of the oil [33]. More broadly, reviews on citrus essential oils indicate that post-harvest handling and storage preferentially destabilize oxygenated fractions relative to their hydrocarbon backbones, thereby altering aroma and technological quality [34]. Similar trends have been described in *Thymus daenensis* and *Mentha* spp., where storage, especially at room temperature, reduces oxygenated constituents while increasing terpene hydrocarbons via hydrolysis, membrane deterioration and volatilization [24], and in leafy aromatic species such as *Eucalyptus citriodora*, which exhibit parallel losses of oxygenated volatiles and rises in sesquiterpenes under extended storage [9]. In Damask rose, even short holding periods before distillation lead to losses of oxygenated aromatic alcohols, accompanied by a relative rise in hydrocarbon sesquiterpenes [25], confirming that oxygenated floral signatures are disproportionately sensitive to post-harvest alterations. Finally, studies on other Rosaceae flowers such as *Prunus mume* show that their floral aroma is dominated by benzenoid esters and aldehydes, whose relative abundances strongly control perceived intensity and quality; any post-harvest decline therefore results in a disproportionate sensory downgrade, in agreement with the patterns observed here for ylang-ylang [35].

These mechanisms align with the present results, namely the marked reduction in aromatic and light oxygenated compounds and the concomitant rise in sesquiterpene hydrocarbons after 24 h (Figure A1). Such changes likely arise from respiration-induced heating, micro-anaerobic conditions within flower piles, ester hydrolysis and oxidation of membrane bound volatiles.

From a quality perspective, the evolution of the proportion of light versus heavy oxygenated compounds, at the expanse of the odor-active constituents, demonstrates a negative effect of storage. The decline of benzenoid esters, the oxygenated aromatic constituents that confer the floral, fruity, jasmine-like and, in some cases, sweet and narcotic top notes of ylang-ylang, combined with the relative enrichment of hydrocarbons, suggests a loss of aromatic finesse and increased dryness of the oil, as is similarly observed for rose and lavender oils subjected to delayed distillation [11].

In this regard, esters as the main subgroups of chemical compounds, seem to be influenced by storage duration, as their content decreased after 24 h of storage (Figure A1). Ether oxides also contributed to the total oxygenated compounds and their content was heading towards a decrease during storage, but this evolution was not significant. On the contrary, the terpene content significantly increased during storage from ST8 to ST24. This is also in agreement with previous work on damask rose flower storage, which reported that the concentration of hydrocarbons in rose flowers stored for various durations under room-temperature conditions displayed higher scores than petals distilled immediately [36].

Although several studies have investigated the effects of plant material storage on essential-oil quality, information on the dynamics of secondary metabolites during storage remains limited for some species and specific contexts and is particularly scarce for ylang-ylang. These metabolites are highly volatile and therefore susceptible to alteration under storage conditions. In *Thymus daenensis*, refrigerated or frozen storage slowed, but did not eliminate, three-month compositional drift, whereas room temperature accelerated losses of oxygenated constituents, consistent with hydrolysis, oxidation and volatilization of labile compounds [24]. Flower crop models closely parallel these dynamics. In *Rosa damascena*, both short ambient holding and longer storage altered EO profiles: extended fermentation and holding shifted the balance toward hydrocarbons and away from oxygenated impact compounds, while cold storage (0–4 °C) mitigated yield and compositional losses relative to ambient conditions [26]. These findings align with patterns observed here after more than 8 h of storage. Similar trends have been reported in *Mentha* spp., where post-harvest handling and storage duration strongly influenced essential-oil yield and composition. Extended chilled storage induced changes in key oxygenated constituents and even modified bioactivity, suggesting that biochemical (hydrolysis, oxidation) and physical (volatilization, membrane alteration) processes occurring during storage can reshape the aromatic and functional profile of the oils. Therefore, the compositional and yield changes observed in this study likely represent conservative estimates of storage-induced alterations. Under typical production conditions involving bagging or piling of flowers, higher internal temperatures and reduced aeration may exacerbate thermal and fermentative processes, potentially leading to more pronounced quality degradation. Altogether, this emphasizes that a minimal storage period before distillation is essential to preserve oxygenated fractions and overall oil fragrance.

### 3.4. Evolution of Essential-Oil Yield According to Harvest Time

Ylang-ylang essential-oil yield was significantly influenced by the time of flower harvesting (Table 1). The highest yields were recorded for flowers harvested at 07:00 a.m. (HT7) and 3:00 p.m. (HT15), while the lowest yield was obtained at 11:00 a.m. (HT11). This pattern indicates a diurnal modulation of volatile accumulation and loss, suggesting that abiotic factors shape the extractable oil fraction within a single day (Table 1).

Comparable trends have been reported in other essential-oil crops, particularly in aromatic flowers, where higher yields coincide with early harvests or periods of limited thermal stress. For instance, Mosleh Arani et al. observed that Damask rose petals produced significantly higher essential-oil yields when harvested during cooler periods, whereas later collection under warmer conditions resulted in substantial losses. Similarly, Jamoussi et al. demonstrated that the essential-oil yield of *Matricaria recutita* (German chamomile) was maximized at early flowering harvests, confirming that essential-oil accumulation responds strongly to short term temporal and environmental cues [37,38]. In addition to yield variations, essential oils also undergo diurnal changes in composition, indicating a dynamic adjustment of secondary metabolism across the day–night cycle. Experimental evidence shows that fluctuations in temperature and light can alter volatile abundance and profiles within short time intervals [39].

The superior yields observed at HT7 can be attributed to physiological conditions prevailing during the night and early morning. In aromatic species, harvest time is a critical determinant of essential-oil content and composition, largely due to fluctuations in temperature, transpiration and metabolic activity across the photoperiod [5,29]. Early-morning harvesting benefits from cooler temperatures and reduced volatilization, favoring the retention of terpenes and benzenoid esters in floral tissues.

Around midday, elevated temperatures and peak transpiration favor the volatilization and possible metabolic transformation of stored aroma compounds, resulting in decreased oil recovery, an effect similarly documented in *Thymbra spicata* [5] and in the aromatic species *Thymus vulgaris* and *Mentha pulegium* [40] when harvested under warmer conditions. The lower yield observed at 11:00 a.m. likely reflects late-morning physiological and radiative conditions, characterized by increasing solar radiation, elevated transpiration rates, and rapidly rising floral surface temperatures, rather than the absolute daily temperature maximum.

Conversely, the recovery of yield at HT15 suggests a partial re-equilibration phase later in the afternoon, when thermal stress declines and metabolic activity rises again, a pattern previously noted in flower crops such as *Matricaria recutita*, which exhibit bimodal diurnal fluctuations in volatile biosynthesis and accumulation [38].

Taken together, these results confirm that harvest timing is crucial for optimizing ylang-ylang oil yield. Early morning (HT7), and to a lesser extent late afternoon (HT15), offer the most favorable conditions for maximizing production, whereas midday harvesting (HT11) should be avoided due to heat-induced volatilization losses.

### 3.5. Evolution of Chemical Compounds Families According to Harvest Time

**Figure A2:**
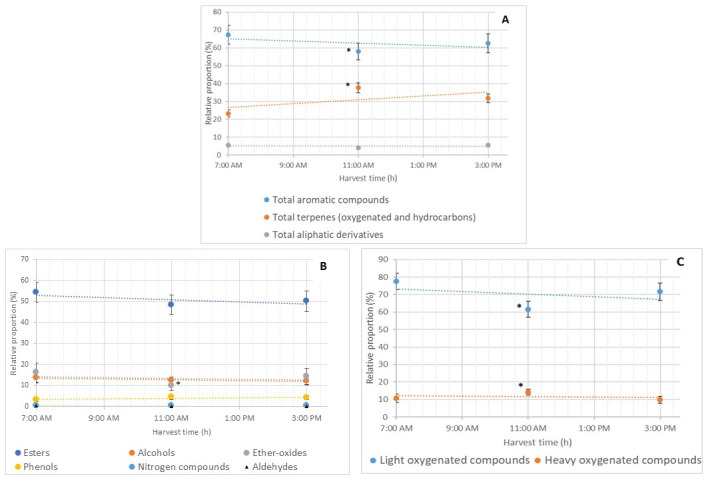
Evolution of chemical compound family (group) proportion according to harvest time of ylang-ylang flowers. (**A**)—families of chemical compounds, (**B**)—subgroups of chemical compounds, (**C**)—oxygenated compounds. Values are presented as mean ± SD, n = 3. A significant difference with the earliest distillation for the same family of chemical compounds is indicated with an asterisk: *, *p* < 0.05.

None of the three major families of chemical compounds exhibited a statistically significant response to harvest time; however, consistent compositional trends were observed (Figure A2). The relative proportion of total aromatic compounds tended to be higher at HT7 (67.32 ± 5.25%) and HT15 (62.51 ± 5.33%) and lower at HT11 (57.92 ± 4.65%). In contrast, total terpene content showed an opposite tendency, with higher values at HT11 (37.76 ± 2.74%) compared with HT7 (23.30 ± 1.95%) and HT15 (31.84 ± 2.31%). This inversion reflects a shift in the balance between the aromatic and terpene fractions around midday, without constituting statistically distinct compositional profiles (Figure A2).

Similar associations between hydrocarbon enrichment and the depletion of oxygenated aroma-impacting compounds and a loss of sensory value have been reported in *Rosa damascena*, *Thymus daenensis*, *Mentha* spp. and *Eucalyptus citriodora* under conditions promoting volatile depletion or biochemical alteration [7,10,24,26].

Aliphatic derivatives were comparatively stable across harvest times (5.6 ± 0.78% at HT7, 4.08 ± 0.56% at HT11, and 5.5 ± 0.76% at HT15), reflecting a limited sensitivity to diurnal variation. Overall, these compositional patterns delineate two distinct chemotypes: HT7 and HT15, enriched in aromatic volatiles and aligned with superior olfactory profiles, and HT11, characterized by reduced aromatic content and higher terpene levels, indicative of diminished essential-oil quality (Figure A2).

### 3.6. Evolution of Subgroups of Chemical Compounds According to Harvest Time

Light oxygenated compounds dominated the oxygenated fraction at all harvest times, but their abundance differed markedly according to flower collection time. The highest proportions were recorded at HT7 (77.58 ± 4.81%) and HT15 (71.65 ± 4.81%), while HT11 exhibited a significant reduction (61.60 ± 4.13%) (Figure A2). This midday depletion mirrors the compositional trend previously observed at the chemical compound family level and reflects a disruption of the oxygenated fraction during the warmest period of the day, a phenomenon also reported for other aromatic plants where diurnal metabolic shifts alter volatile abundance [41,42].

In contrast, heavy oxygenated compounds followed the opposite pattern: they were most abundant at HT11 (14.25 ± 1.67%) and significantly lower at HT7 (10.76 ± 1.36%) and HT15 (9.78 ± 1.19%) (Figure A2). This inversion suggests a midday shift in oxygenated chemistry, favoring the accumulation or persistence of heavier, less volatile oxygenated structures when lighter oxygenated volatiles are lost. A more detailed breakdown of oxygenated subgroups confirms this dichotomy, consistent with recent evidence showing that the timing of flower collection modulates the distribution of oxygenated subclasses in essential-oil-bearing species [43].

Esters, which constitute the most important contributors to the floral signature of ylang-ylang oil, did not differ significantly across harvest times, though they remained highest at HT7 (54.33 ± 4.69%) and lowest at HT11 (48.53 ± 4.62%). Alcohols followed a similar profile with minor fluctuations, showing no significant sensitivity to harvest timing. In contrast, ether oxides were strongly affected: their proportion was almost halved at HT11 (9.89 ± 2.27%) compared with HT7 (16.17 ± 4.73%) and HT15 (14.50 ± 3.86%), confirming their vulnerability during midday conditions (Figure A2). Similar temporal shifts in oxygenated fractions have also been documented in other essential-oil-bearing crops, where diurnal variation modulated both the light and heavy oxygenated subgroups [44].

Phenols and nitrogen compounds remained stable across harvest times, suggesting robust metabolic regulation. Aldehydes, however, showed a pronounced decline from HT7 (0.36 ± 0.05%) to HT11 (0.14 ± 0.03%) and HT15 (0.09 ± 0.02%), indicating rapid conversion or volatilization dynamics during and after peak daytime temperatures (Figure A2).

Taken together, these results reveal two distinct temporal chemotypes based on oxygenated subgroup behavior: HT7 and HT15 share similar profiles dominated by light, oxygenated, aroma-defining compounds, preserving floral quality markers. HT11 diverges significantly, characterized by depletion of light oxygenated volatiles and relative enrichment of heavy oxygenated fractions and ether-oxide losses, consistent with a midday compositional shift detrimental to olfactory performance.

### 3.7. Evolution of Volatile Compounds According to Harvest Time

Harvest time markedly affected the abundance of several key volatile constituents identified in ylang-ylang essential oil, with the most compositional variations occurring between 07:00 a.m. (HT7) and 11:00 a.m. (HT11), and partial re-equilibration at 03:00 p.m. (HT15) (Table 2). This trend is consistent with previous observations reporting that diurnal harvesting windows markedly influence both the yield and chemical composition of floral and leafy essential oils in several aromatic species, including sage, thyme, rosemary and other medicinal plants [40,41,45,46].

Among the benzenoid esters, benzyl acetate, the most abundant compound, showed relatively stable levels between HT7 (19.66%) and HT15 (20.78%), but experienced a slight decrease at HT11 (19.18%). Methyl benzoate followed a similar pattern, with higher proportions at HT7 (8.96%) and HT15 (9.66%) compared with HT11 (8.04%) (Table 2), confirming its susceptibility to temperature-driven volatilization during late-morning hours, in agreement with studies showing that early-morning harvests help preserve more volatile oxygenated fractions before midday warming reduces their abundance [5,9,41]. In contrast, benzyl benzoate and benzyl salicylate behaved antagonistically: both increased at HT11 (6.60% and 2.79%, respectively) before decreasing again at HT15 (4.78% and 1.75%). These two heavier compounds appear to accumulate or persist when lighter esters decline, explaining their relative enrichment at midday, a dynamic also observed in other essential-oil bearing flowers where heavier hydrocarbons temporarily dominate under warmer conditions [9,47]. Prenyl benzoate exhibited a distinct profile, being detected only at HT7 (4.14%) and absent at both HT11 and HT15, suggesting synthesis or accumulation during early-morning metabolic activity followed by rapid degradation or volatilization once temperatures rise. Phenyl ethyl acetate, by contrast, maintained nearly identical quantities across all harvest times, confirming its relative stability within the diurnal widow (Table 2), a behavior also noted for stable impact compounds in other aromatic crops during harvest-time comparisons [45].

For ether-linked volatiles, p-cresyl methyl ether showed a pronounced decline at HT11 (9.38%) compared with HT7 (15.47%), before increasing again at HT15 (13.91%). This reversible behavior indicates a thermal sensitivity threshold around midday and a partial recovery later in the afternoon. A comparable dynamic was observed for p-cresol, which was highest at HT7 (0.24%), decreased at HT11 (0.25%), and increased again at HT15 (0.30%) (Table 2), supporting the hypothesis of a diurnal regulation rather than irreversible degradation, consistent with the hour-dependent fluctuations reported in sage essential-oil profiles [41].

Several aldehydes displayed clear midday depletion. Benzeneacetaldehyde, responsible for green floral nuances, was highest at HT7 (0.26%) and drastically reduced at both HT11 (0.09%) and HT15 (0.06%). Benzaldehyde followed a similar trajectory but remained detectable at all three harvest times. The presence of traces of (E)-cinnamaldehyde only at HT11 suggests restricted biosynthesis and low thermal stability under morning and afternoon conditions (Table 2), which aligns with the behavior of the harvest-sensitive aldehydes reported in Tagetes minuta and other aromatic species [46].

Harvest time also affected the abundance of heavier volatiles. α-Humulene presented a marked surge at HT11 (11.02%) before declining at HT15 (8.60%), marking HT11 as a compositional inflection point where lighter constituents diminish and heavier ones transiently dominate. Germacrene-D, δ-cadinene, α-copaene and α-farnesene all followed this transient rise at HT11 before decreasing again at HT15, demonstrating that their midday enrichment likely reflects proportional accumulation associated with the depletion of more volatile compounds (Table 2), similarly to trends reported in other sesquiterpene-rich species [41,47].

Conversely, linalool, one of the few oxygenated alcohols quantified individually, was maximal at HT7 (9.37%), declined at HT11 (6.35%) and rose again at HT15 (7.75%). Geraniol displayed the opposite trend, with its highest level at HT11 (0.33%) before falling at HT15 (0.10%), indicating a midday enhancement of the corresponding metabolic pathway (Table 2), a behavior consistent with observations that oxygenated monoterpenes fluctuate with harvest time in rosemary and other aromatic taxa [36,40].

Taken together, these compositional trajectories demonstrate that HT7 oils concentrate the most desirable floral signatures (benzyl acetate, methyl benzoate, p-cresyl methyl ether, benzeneacetaldehyde, linalool), HT11 oils show a depletion of these highly volatile constituents and a temporary dominance of heavier molecules (benzyl benzoate, benzyl salicylate, α-humulene, germacrene-D), while HT15 oils partially recover morning attributes without fully restoring the chemical freshness observed at HT7, in agreement with broader findings from diurnal harvest studies on other essential-oil-producing plants [40,41].

Ylang-ylang essential oil represents a strategic raw material for the fragrance, cosmetic, detergent, and soap industries, as well as for aromatherapy applications. It is widely used in the production of luxury perfumes, personal-care products, scented household formulations, and wellness products. Its market value strongly depends on its chemical composition, sensory attributes, and compliance with international quality standards. Therefore, controlling post-harvest and storage conditions is essential to ensure consistent quality, industrial suitability, and commercial competitiveness.

## 4. Conclusions

This study shows that both flower storage duration and harvest time are key determinants of the yield and chemical quality of ylang-ylang essential oil. The essential-oil yield and composition were preserved when flowers were distilled within 8 h after harvest, whereas prolonged storage (24–30 h) caused significant yield losses and pronounced compositional shifts, marked by a decline in aromatic and light oxygenated compounds and a relative increase in terpene hydrocarbons and heavy oxygenated fractions. Harvest timing also influenced oil performance, with early morning (07:00 a.m.) and late afternoon (3:00 p.m.) harvests producing a higher yield of oils and more favorable chemical profiles than midday harvesting (11:00 a.m.). Based on these results obtained under controlled storage conditions, ylang-ylang flowers should be harvested during cooler periods of the day and distilled as rapidly as possible, preferably within 8 h after harvest, to minimize post-harvest degradation and preserve the fragrance that underpin essential-oil quality. Under practical field conditions involving compression and limited aeration, post-harvest losses may be even more pronounced.

## Figures and Tables

**Table 1:** Weight loss of ylang-ylang flowers during open-air shaded storage in a shed and essential-oil yields (expressed on an initial-fresh-weight basis) according to storage duration and harvest time.

	Storage Duration					Harvest Time		
	ST0	ST4	ST8	ST24	ST30	HT7	HT11	HT15
Flower weight loss (%)	0 ^a^	21.56 ± 4.2 ^b^	33.56 ± 3.7 ^b^	61.93 ± 2.1 ^c^	69.63 ± 2.3 ^c^	-	-	-
Essential-oil yield (%)	0.78 ± 0.1 ^a^	0.76 ± 0.12 ^a^	0.76 ± 0.1 ^a^	0.73 ± 0.09 ^b^	0.72 ± 0.07 ^b^	0.81 ± 0.14 ^a^	0.77 ± 0.11 ^b^	0.80 ± 0.15 ^a^

(ST0 = 0 h, ST4 = 4 h, ST8 = 8 h, ST24 = 24 h, ST30 = 30 h flower storage before distillation; HT7 = 7:00 a.m., HT11 = 11:00 a.m., HT15 = 3:00 p.m. flower harvest time). Values are mean ± standard deviation of the mean and were averages of three extractions of each type of method. Values followed by a different letter within the lines are significantly different (*p* ≤ 0.05), Tukey test.

**Table 2:** Chemical composition of ylang-ylang essential oils as affected by flower storage duration and harvest time.

	RT (min)	RIs	Compound	Storage Duration					Harvest Time		
				ST0	ST4	ST8	ST24	ST30	HT7	HT11	HT15
**Subgroups**											
Ester	12.11	1195	methyl benzoate	9.25 ± 1.02 ^a^	8.76 ± 2.22 ^a^	8.82 ± 1.71 ^a^	3.64 ± 0.63 ^b^	3.77 ± 1.26 ^b^	8.96 ± 1.69 ^a^	8.04 ± 1.42 ^a^	9.66 ± 1.15 ^a^
	12.98	1248	benzyl acetate	27.87 ± 1.97 ^a^	25.68 ± 4.6 ^a^	24.79 ± 3.42 ^a^	10.8 ± 1.45 ^b^	10.91 ± 1.28 ^b^	19.66 ± 3.01 ^a^	19.18 ± 3.89 ^a^	20.78 ± 3.48 ^a^
	13.17	1260	phenyl ethyl acetate	0.37 ± 0.03 ^a^	0.37 ± 0.09 ^a^	0.41 ± 0.11 ^a^	0.31 ± 0.09 ^a^	0.33 ± 0.07 ^a^	0.27 ± 0.03 ^a^	0.28 ± 0.08 ^a^	0.24 ± 0.08 ^a^
	13.60	1286	ethyl benzoate	0.02 ± 0.01 ^a^	0.03 ± 0.01 ^a^	0.06 ± 0.02 ^b^	0.12 ± 0.03 ^c^	0.16 ± 0.05 ^c^	0.07 ± 0.02 ^a^	0.08 ± 0.03 ^a^	0.09 ± 0.02 ^a^
	13.69	1292	methyl salicylate	0.31 ± 0.03 ^a^	0.3 ± 0.03 ^a^	0.33 ± 0.04 ^a^	0.23 ± 0.05 ^a^	0.28 ± 0.07 ^a^	0.31 ± 0.06 ^a^	0.34 ± 0.05 ^a^	0.3 ± 0.02 ^a^
	14.56	1345	benzoicacid, 4-methoxy, methyl ester	0.08 ± 0 ^a^	0.02 ± 0.02 ^b^	0.11 ± 0.02 ^a^	0.11 ± 0.02 ^a^	0.12 ± 0.02 ^a^	-	0.06 ± 0.02 ^a^	0.04 ± 0.01 ^a^
	14.87	1364	3-phenyl-1-propanol acetate	0.03 ± 0 ^a^	-	0.03 ± 0.01 ^a^	0.03 ± 0 ^a^	0.03 ± 0.01 ^a^	-	0.02 ± 0.03 ^a^	-
	16.36	1455	(E)-cinnamyl acetate	7.75 ± 0.26 ^a^	6.99 ± 0.97 ^a^	7.64 ± 0.57 ^a^	8.54 ± 0.88 ^a^	7.67 ± 1.23 ^a^	5.96 ± 1.44 ^a^	5.63 ± 1.06 ^a^	5.73 ± 0.96 ^a^
	16.50	1463	prenyl benzoate	-	-	1.07 ± 0.33 ^ab^	1.63 ± 0.24 ^b^	1.32 ± 0.19 ^ab^	4.14 ± 1.1 ^a^	-	-
	17.10	1500	4-methylpentanoic acid, benzyl ester	-	-	-	0.05 ± 0.02 ^a^	0.04 ± 0.02 ^a^	0.02 ± 0.01 ^a^	-	-
	18.2	1569	3-hexen-1-ol, benzoate, (Z)	0.17 ± 0.01 ^a^	0.15 ± 0.06 ^a^	0.16 ± 0.03 ^a^	0.12 ± 0.03 ^a^	0.15 ± 0.05 ^a^	0.03 ± 0.02 ^a^	-	-
	21.2	1766	benzyl benzoate	7.7 ± 1.4 ^a^	8.85 ± 0.84 ^a^	8.12 ± 1.49 ^a^	14.79 ± 1.3 ^b^	12.81 ± 2.61 ^b^	5.44 ± 1.83 ^a^	6.6 ± 0.7 ^a^	4.78 ± 1.54 ^a^
	22.3	1844	benzyl salicylate	3.34 ± 0.6 ^a^	4.43 ± 0.4 ^a^	3.67 ± 0.72 ^a^	8.26 ± 1.13 ^b^	6.5 ± 1.53 ^b^	2.13 ± 0.2 ^a^	2.79 ± 0.3 ^a^	1.75 ± 0.55 ^a^
Phenol	10.30	1087	p-cresol	0.37 ± 0.03 ^a^	0.31 ± 0.06 ^ab^	0.31 ± 0.07 ^ab^	0.2 ± 0.06 ^b^	0.18 ± 0.03 ^b^	0.24 ± 0.07 ^a^	0.25 ± 0.04 ^a^	0.3 ± 0.09 ^a^
	11.54	1161	creosol	0.04 ± 0.01	0.04 ± 0.01	0.05 ± 0.01	0.04 ± 0.01	0.03 ± 0.01	-	-	-
	13.10	1256	chavicol	0.13 ± 0.01 ^a^	-	-	-	-	-	-	-
	16.38	1456	*trans*-isoeugenol	3.51 ± 0.36 ^a^	3.67 ± 1.07 ^a^	3.6 ± 0.85 ^a^	3.74 ± 0.38 ^a^	4.09 ± 1.1 ^a^	3.09 ± 1.21 ^a^	4.27 ± 0.84 ^a^	3.97 ± 0.22 ^a^
Ether oxide	9.30	1026	p-cresyl methyl ether	11.56 ± 1.27 ^a^	9.92 ± 2.9 ^a^	11.18 ± 2.75 ^a^	6.23 ± 1.62 ^b^	7.02 ± 1.83 ^b^	15.47 ± 3.74 ^a^	9.38 ± 3.41 ^a^	13.91 ± 2.81 ^a^
	11.3	1146	1,2-Dimethoxybenzene	0.02 ± 0 ^a^	0.03 ± 0.02 ^a^	0.03 ± 0.01 ^a^	0.01 ± 0.01 ^a^	0.01 ± 0.01 ^a^	-	-	-
	13.5	1280	anethole	0.45 ± 0.04 ^a^	0.45 ± 0.06 ^a^	0.57 ± 0.08 ^a^	0.56 ± 0.08 ^a^	0.72 ± 0.16 ^a^	0.65 ± 0.09 ^a^	0.5 ± 0.13 ^a^	0.55 ± 0.09 ^a^
Nitrogen compound	11.3	1143	phenylacetonitrile	-	0.06 ± 0.02 ^a^	0.08 ± 0.02 ^a^	0.07 ± 0.01 ^a^	0.09 ± 0.01 ^a^	0.08 ± 0.01 ^a^	0.05 ± 0.05 ^b^	0.1 ± 0.02 ^a^
	11.4	1152	benzene, 1-isocyano-2-methyl	-	-	-	0.01 ± 0.01 ^a^	0.01 ± 0 ^a^	0.06 ± 0.03 ^a^	0.01 ± 0.01 ^b^	-
	15.44	1399	2-phenyl nitroethane	0.4 ± 0 ^a^	0.37 ± 0.06 ^a^	0.45 ± 0.06 ^a^	0.38 ± 0.14 ^a^	0.33 ± 0.03 ^a^	0.41 ± 0.2	0.27 ± 0.1 ^a^	0.22 ± 0.04 ^a^
Aldehyde	8.70	988	benzaldehyde	-	0.06 ± 0.03 ^a^	0.07 ± 0.02 ^ab^	0.13 ± 0.01 ^bc^	0.16 ± 0.04 ^c^	0.07 ± 0.01 ^a^	0.05 ± 0 ^b^	0.03 ± 0.01 ^b^
	9.6	1044	benzeneacetaldehyde	0.29 ± 0.01 ^a^	0.19 ± 0.06 ^ab^	0.17 ± 0.07 ^ab^	0.12 ± 0.03 ^b^	0.15 ± 0.05 ^b^	0.26 ± 0.07 ^a^	0.09 ± 0.02 ^b^	0.06 ± 0.02 ^b^
	13.33	1270	cinnamaldehyde, (E)	-	0.07 ± 0.02 ^a^	0.04 ± 0.07 ^a^	0.14 ± 0.01 ^b^	0.14 ± 0.05 ^b^	-	0.03 ± 0.02 ^a^	-
Monoterpene hydrocarbon	7.9	934	α-pinene	0.06 ± 0.01 ^a^	0.05 ± 0.02 ^a^	0.07 ± 0.01 ^a^	0.09 ± 0.02 ^a^	0.11 ± 0.02 ^a^	0.06 ± 0.02 ^a^	0.06 ± 0.01 ^a^	0.06 ± 0.01 ^a^
	8.7	988	β-myrcene	0.03 ± 0 ^a^	-	-	-	-	-	-	-
	10.21	1081	isoterpinolene	-	-	-	-	-	-	0.03 ± 0.03 ^a^	0.04 ± 0.01 ^a^
Sesquiterpene hydrocarbon	14.4	1337	δ-elemene	-	0.1 ± 0.03 ^a^	0.11 ± 0.03 ^a^	0.13 ± 0.01 ^a^	0.17 ± 0.01 ^a^	0.13 ± 0.04 ^a^	0.03 ± 0.01 ^b^	0.32 ± 0.04 ^c^
	14.66	1351	α-cubenene	0.07 ± 0.01 ^a^	0.09 ± 0.03 ^ab^	0.09 ± 0.02 ^ab^	0.14 ± 0.04 ^b^	0.16 ± 0.04 ^b^	0.04 ± 0.02 ^a^	0.03 ± 0.01 ^ab^	0.01 ± 0.01 ^b^
	15.05	1375	α-ylangene	-	0.1 ± 0.02 ^a^	-	-	-	0.1 ± 0.02 ^a^	0.03 ± 0.01 ^b^	0.11 ± 0.02 ^a^
	15.1	1380	α-copaene	0.15 ± 0.03 ^a^	0.22 ± 0.09 ^ab^	0.2 ± 0.14 ^ab^	0.34 ± 0.08 ^bc^	0.41 ± 0.13 ^c^	0.16 ± 0.05 ^a^	0.28 ± 0.1 ^b^	0.2 ± 0.04 ^ab^
	16.0	1434	aromadendrene	0.16 ± 0.03 ^a^	0.25 ± 0.1 ^a^	0.26 ± 0.1 ^a^	-	-	-	0.3 ± 0.26 ^a^	-
	16.35	1454	α-humulene	0.18 ± 0.02 ^ab^	1.07 ± 0.1 ^a^	0.23 ± 0.08 ^b^	0.52 ± 0.06 ^c^	1.02 ± 0.45 ^a^	0.23 ± 0.05 ^a^	11.02 ± 1.18 ^b^	8.6 ± 0.93 ^b^
	16.46	1461	alloaromadendrene	-	-	-	0.43 ± 0.01 ^a^	0.49 ± 0.09 ^a^	0.3 ± 0.1 ^a^	-	-
	16.77	1480	germacrene-D	5.06 ± 0.96 ^a^	6.26 ± 1.34 ^a^	6.46 ± 1.4 ^a^	10.38 ± 0.96 ^b^	11.26 ± 2.19 ^b^	1.23 ± 0.2 ^a^	1.85 ± 0.23 ^a^	1.43 ± 0.16 ^a^
	16.85	1485	zonarene	0.05 ± 0.01 ^ab^	0.07 ± 0.02 ^ab^	0.08 ± 0.03 ^bc^	0.04 ± 0.02 ^a^	0.13 ± 0.04 ^c^	0.06 ± 0.02 ^a^	0.05 ± 0.01 ^a^	0.04 ± 0.01 ^a^
	17.27	1511	(E,E)-α-farnesene	5.53 ± 1.02 ^a^	6.12 ± 1.49 ^a^	5.43 ± 1.98 ^a^	8.93 ± 1.17 ^b^	9.29 ± 2.51 ^b^	5.49 ± 1.8 ^a^	8.98 ± 1.32 ^a^	6.68 ± 0.82 ^a^
	17.45	1522	α-cadinene	-	-	0.09 ± 0.09 ^a^	0.13 ± 0.1 ^a^	0.15 ± 0.1 ^a^	-	0.04 ± 0.01 ^a^	-
	17.48	1524	δ-cadinene	0.64 ± 0.1 ^a^	0.94 ± 0.22 ^ab^	0.86 ± 0.24 ^ab^	1.34 ± 0.16 ^b^	1.28 ± 0.4 ^b^	0.02 ± 0.01 ^a^	1.11 ± 0.49 ^b^	0.89 ± 0.1 ^b^
Sesquiternene alcohol	10.7	1110	linalool	6.3 ± 0.58 ^a^	5.02 ± 1.32 ^a^	6.03 ± 0.97 ^a^	6.03 ± 1.51 ^a^	7.31 ± 2.41 ^a^	9.37 ± 1.81 ^a^	6.35 ± 0.61 ^a^	7.75 ± 1.48 ^a^
	12.64	1227	nerol	-	-	-	0.11 ± 0.03 ^a^	0.03 ± 0.02 ^b^	0.01 ± 0.01 ^a^	0.02 ± 0.01 ^a^	-
	13.16	1259	geraniol	-	0.02 ± 0.01 ^a^	0.02 ± 0.01 ^a^	0.06 ± 0.02 ^b^	0.06 ± 0.02 ^b^	0.15 ± 0.03 ^a^	0.33 ± 0.06 ^b^	0.1 ± 0.03 ^a^
	17.34	1515	cubebol	0.06 ± 0.02 ^a^	-	-	0.18 ± 0.03 ^b^	0.25 ± 0.07 ^b^	-	-	-
	17.88	1549	α-elemol	0.06 ± 0.01 ^a^	0.02 ± 0.01 ^b^	0.06 ± 0.02 ^a^	0.12 ± 0.03 ^c^	0.09 ± 0.04 ^a^	0.01 ± 0.01 ^a^	0.04 ± 0.01 ^b^	0.01 ± 0.01 ^a^
	18.09	1562	trans-nerolidol	-	0.06 ± 0.02 ^a^	-	-	-	0.03 ± 0.02 ^a^	0.01 ± 0.01 ^a^	-
	18.28	1574	germacrene-D-4-ol	-	-	-	-	0.12 ± 0.09 ^a^	0.06 ± 0.03 ^a^	-	0.07 ± 0.03 ^a^
	18.72	1602	guaiol	0.21 ± 0.01 ^a^	0.34 ± 0.08 ^ab^	0.32 ± 0.07 ^ab^	0.56 ± 0.12 ^bc^	0.66 ± 0.21 ^c^	0.05 ± 0.02 ^a^	0.11 ± 0.03 ^b^	0.07 ± 0.05 ^ab^
	18.97	1618	junenol	-	0.22 ± 0.07 ^a^	0.1 ± 0.03 ^b^	-	-	0.15 ± 0.03 ^a^	0.67 ± 0.07 ^b^	0.2 ± 0.07 ^a^
	19.06	1624	10-epi-γ-eudesmol	-	0.1 ± 0.03 ^a^	0.09 ± 0.01 ^a^	-	-	0.01 ± 0.01 ^a^	0.09 ± 0.03 ^b^	0.01 ± 0 ^a^
	19.08	1625	caryophyllene alcohol	0.14 ± 0.03 ^a^	-	-	-	-	0.12 ± 0.03 ^a^	0.12 ± 0.04 ^a^	0.05 ± 0.02 ^b^
	19.20	1633	β-acorenol	0.09 ± 0.01 ^a^	0.13 ± 0.04 ^ab^	0.12 ± 0.04 ^ab^	0.23 ± 0.05 ^b^	0.23 ± 0.1 ^b^	0.06 ± 0.03 ^a^	-	-
	19.31	1640	τ-cadinol	0.78 ± 0.17 ^a^	1.28 ± 0.83 ^b^	0.9 ± 0.33 ^a^	1.37 ± 0.24 ^b^	1.37 ± 0.3 ^b^	0.73 ± 0.23 ^a^	1.16 ± 0.32 ^a^	0.87 ± 0.13 ^a^
	19.34	1642	epi-cubenol	-	0.07 ± 0.02 ^ab^	0.06 ± 0.05 ^a^	0.13 ± 0.02 ^bc^	0.16 ± 0.06 ^c^	0.07 ± 0.03 ^a^	0.16 ± 0.04 ^b^	0.09 ± 0.04 ^ab^
	19.38	1645	τ-muurolol	0.72 ± 0.16 ^a^	1.26 ± 0.33 ^b^	0.85 ± 0.35 ^a^	1.25 ± 0.25 ^b^	1.31 ± 0.34 ^b^	0.8 ± 0.29 ^a^	0.7 ± 0.16 ^a^	0.86 ± 0.17 ^a^
	20.55	1722	(E,E)-farnesol	1.24 ± 0.23 ^a^	1.46 ± 0.32 ^a^	1.38 ± 0.32 ^a^	2.48 ± 0.27 ^b^	2.24 ± 0.58 ^ab^	1.97 ± 0.21 ^a^	2.6 ± 0.46 ^a^	1.9 ± 0.42 ^a^
Monoterpene ester	15.15	1381	geranyl acetate	0.88 ± 0.04 ^a^	0.77 ± 0.08 ^a^	0.99 ± 0.12 ^a^	1.24 ± 0.16 ^a^	1.37 ± 0.19 ^ba^	1.84 ± 0.07 ^a^	1.58 ± 0.51 ^a^	1.44 ± 0.3 ^a^
Monoterpene oxide	9.41	1033	eucalyptol	0.06 ± 0.01 ^a^	-	0.04 ± 0.04 ^a^	0.05 ± 0.02 ^a^	0.05 ± 0.04 ^a^	0.05 ± 0.02 ^a^	0.01 ± 0.01 ^b^	0.04 ± 0.02 ^a^
Ester	6.44	812	butyl acetate	0.1 ± 0.01 ^a^	-	-	-	-	-	-	-
	6.98	861	3-methyl-3-butene-1-ol acetate	0.79 ± 0.1 ^a^	0.63 ± 0.31 ^a^	0.71 ± 0.27 ^a^	0.23 ± 0.09 ^b^	0.2 ± 0.06 ^b^	1.55 ± 0.45 ^a^	0.81 ± 0.16 ^a^	1.37 ± 0.41 ^a^
	7.67	910	prenyl acetate	1.05 ± 0.03 ^a^	0.88 ± 0.26 ^a^	1.07 ± 0.31 ^a^	0.41 ± 0.14 ^b^	0.4 ± 0.1 ^b^	2.25 ± 0.4 ^a^	0.78 ± 0.15 ^b^	2.29 ± 0.49 ^a^
	9.02	1009	*cis*-3-hexenyl-acetate	0.17 ± 0.01 ^a^	0.15 ± 0.06 ^a^	0.16 ± 0.03 ^a^	0.12 ± 0.03 ^a^	0.15 ± 0.05 ^a^	0.13 ± 0.02 ^a^	0.09 ± 0.05 ^a^	0.02 ± 0.02 ^b^
	9.07	1012	hexyl acetate	0.21 ± 0.05 ^a^	0.18 ± 0.07 ^a^	0.23 ± 0.05 ^a^	0.21 ± 0.05 ^a^	0.22 ± 0.1 ^a^	0.46 ± 0.14 ^a^	0.25 ± 0.08 ^a^	0.45 ± 0.11 ^a^
	21.76	1804	(E,E)-farnesyl acetate	0.73 ± 0.22 ^a^	1.05 ± 0.35 ^ab^	0.78 ± 0.31 ^a^	1.96 ± 0.45 ^b^	1.64 ± 0.34 ^b^	0.97 ± 0.14 ^a^	1.75 ± 0.19 ^a^	0.92 ± 0.3 ^a^
Alcohol	5.36	603	2-methyl-3-butene-2-ol	0.06 ± 0.02 ^a^	0.07 ± 0.02 ^a^	0.1 ± 0.03 ^a^	0.05 ± 0.01 ^a^	0.08 ± 0.01 ^a^	0.14 ± 0.04 ^a^	0.25 ± 0.1 ^b^	0.29 ± 0.06 ^b^
	6.20	778	prenol	-	-	0.04 ± 0.02 ^a^	0.01 ± 0.01 ^b^	0.02 ± 0.01 ^a^	0.07 ± 0.02 ^a^	0.06 ± 0.01 ^a^	0.12 ± 0.03 ^b^
	20.51	1719	2-methyl-4-(2,6,6-trimethylcyclohex-1-enyl)but-2-en-1-ol	-	-	-	0.05 ± 0.01 ^a^	0.05 ± 0.02 ^a^	-	-	-
Aldehyde	5.85	704	pentanal	0.03 ± 0.01 ^a^	0.03 ± 0.02 ^a^	0.04 ± 0.01 ^a^	0.04 ± 0.02 ^a^	0.04 ± 0.01 ^a^	0.03 ± 0	-	-
	6.09	854	*trans*-2-hexenal	0.19 ± 0.01 ^a^	0.1 ± 0.04 ^ab^	0.1 ± 0.05 ^ab^	0.06 ± 0.01 ^b^	0.05 ± 0.02 ^b^			
	7.48	905	heptanal	-	-	-	0.03 ± 0.01 ^a^	0.03 ± 0.01 ^a^	-	-	-
Alkane	6.0	735	hexane, 2,4-dimethyl	0.03 ± 0.01 ^a^	0.05 ± 0.03 ^a^	0.05 ± 0.02 ^a^	0.06 ± 0.02 ^a^	0.06 ± 0.03 ^a^	-	-	-
	6.31	800	octane	-	-	-	-	-	-	0.06 ± 0.03 ^a^	-
Ketone	8.68	987	6-methylhept-5-en-2-one	0.03 ± 0 ^a^	0.03 ± 0.01 ^a^	0.04 ± 0.01 ^a^	0.05 ± 0.01 ^a^	0.06 ± 0.01 ^a^	-	-	-
	20.74	1735	cyclopentanone, 3-[3,5-decadienyl], (Z,Z)	-	-	-	0.04 ± 0.01 ^a^	0.04 ± 0.02 ^a^	-	-	-
**Families**											
Total aromatic compounds				73.66 ± 6.10 ^a^	70.75 ± 5.76 ^a^	71.76 ± 5.56 ^a^	60.26 ± 4.03 ^b^	57.02 ± 3.70 ^b^	67.32 ± 5.25 ^a^	57.92 ± 4.65 ^a^	62.51 ± 5.33 ^a^
Total terpenes				22.47 ± 1.65 ^a^	26.02 ± 1.69 ^a^	24.84 ± 1.74 ^a^	36.28 ± 2.52 ^b^	39.72 ± 2.73 ^b^	23.3 ± 1.95 ^a^	37.76 ± 2.74 ^b^	31.84 ± 2.31 ^ab^
Total aliphatic derivatives				3.69 ± 0.45 ^a^	2.54 ± 0.34 ^a^	3.09 ± 0.35 ^a^	3.32 ± 0.49 ^a^	3.04 ± 0.41 ^a^	5.6 ± 0.78 ^a^	4.08 ± 0.56 ^a^	5.5 ± 0.76 ^a^
Total compounds (%)				99.52	99.31	99.69	99.86	99.78	96.22	99.43	99.81

(ST0 = 0 h, ST4 = 4 h, ST8 = 8 h, ST24 = 24 h, ST30 = 30 h flower storage before distillation; HT7 = 7:00 a.m., HT11 = 11:00 a.m., HT15 = 3:00 p.m. flower harvest time). Values are mean ± standard deviation of the mean and were averages of three extractions of each type of method. Values followed by a different letter within the lines are significantly different (*p* ≤ 0.05), Tukey test.

## Data Availability

The original contributions presented in this study are included in the article. Further inquiries can be directed to the corresponding author.
